# Effect of Novel Bioactive Glass-Containing Dentin Adhesive on the Permeability of Demineralized Dentin

**DOI:** 10.3390/ma14185423

**Published:** 2021-09-19

**Authors:** Hyun-Jung Kim, Ji-Hyun Jang, Sang Uk Woo, Kyoung-Kyu Choi, Sun-Young Kim, Jack L. Ferracane, Jung-Hwan Lee, Dongseok Choi, Samjin Choi, Soogeun Kim, Ayoung Bang, Duck-Su Kim

**Affiliations:** 1 Department of Conservative Dentistry, Kyung Hee University Dental Hospital, Seoul 02453, Korea; kimhyunjung@khu.ac.kr; 2 Department of Conservative Dentistry, School of Dentistry, Kyung Hee University, Seoul 02453, Korea; jangjihyun@khu.ac.kr (J.-H.J.); choikkyu@khu.ac.kr (K.-K.C.); choid@ohsu.edu (D.C.); 3 Department of Conservative Dentistry, Graduate School, Kyung Hee University, Seoul 02453, Korea; sanugy@naver.com; 4 Department of Conservative Dentistry, School of Dentistry, Dental Research Institute, Seoul National University, Seoul 03080, Korea; denkim@snu.ac.kr; 5 Department of Restorative Dentistry, School of Dentistry, Oregon Health & Science University, Portland, OR 97201, USA; ferracan@ohsu.edu; 6 Department of Biomaterials Science, College of Dentistry, Dankook University, Cheonan 31116, Chungcheongnam-Do, Korea; ducious@gmail.com; 7 Institute of Tissue Regeneration Engineering (ITREN), Dankook University, Cheonan 31116, Chungcheongnam-Do, Korea; 8 Department of Nanobiomedical Science & BK21 PLUS NBM Global Research Center for Regenerative Medicine Research Center, Dankook University, Cheonan 31116, Chungcheongnam-Do, Korea; 9 UCL Eastman-Korea Dental Medicine Innovation Centre, Cheonan 31116, Chungcheongnam-Do, Korea; 10 Oregon Health & Science University-Portland State University School of Public Health, Oregon Health & Science University, Portland, OR 97239, USA; 11 Department of Biomedical Engineering, College of Medicine, Kyung Hee University, Seoul 02453, Korea; medchoi@khu.ac.kr (S.C.); sigamda@khu.ac.kr (S.K.); ayoung.bang@gmail.com (A.B.)

**Keywords:** bioactive glass, dentin remineralization, dentin permeability, dentinal fluid flow, Raman confocal spectroscopy, field-emission scanning electron microscopy

## Abstract

This study aimed to evaluate the effect of a novel bioactive glass (BAG)-containing dentin adhesive on the permeability of demineralized dentin. Bioactive glass (85% SiO_2_, 15% CaO) was fabricated using the sol-gel process, and two experimental dentin adhesives were prepared with 3 wt% silica (silica-containing dentin adhesive; SCA) or BAG (BAG-containing dentin adhesive; BCA). Micro-tensile bond strength (μTBS) test, fracture mode analysis, field-emission scanning electron microscopy (FE-SEM) analysis of adhesive and demineralized dentin, real-time dentinal fluid flow (DFF) rate measurement, and Raman confocal microscopy were performed to compare SCA and BCA. There was no difference in μTBS between the SCA and BCA (*p* > 0.05). Multiple precipitates were evident on the surface of the BCA, and partial occlusion of dentinal tubules was observed in FE-SEM of BCA-approximated dentin. The DFF rate was reduced by 50.10% after BCA approximation and increased by 6.54% after SCA approximation. Raman confocal spectroscopy revealed an increased intensity of the hydroxyapatite (HA) peak on the dentin surface after BCA application. The novel BAG-containing dentin adhesive showed the potential of both reducing dentin permeability and dentin remineralization.

## 1. Introduction

The demineralization of dentin is usually caused by organic acids from oral bacteria or acid etching during an adhesive procedure, which results in the destruction of the dentin matrix [1]. Once demineralized collagen fibers are exposed, they may be degraded by endogenous matrix metalloproteinases (MMPs) from the dentin that is activated by etching [2]. Ideally, the exposed collagen network should be protected by dentin adhesive to prevent collagen degradation after the bonding procedure. However, it is impossible to encapsulate all the demineralized collagen fibers in the hybrid layer [3]. To remineralize dentin and prevent collagen degradation, various agents such as fluoride, sodium trimetaphosphate, zinc oxide, titanium nanoparticles, chlorhexidine, and benzalkonium chloride have been incorporated into dentin adhesives [4,5,6,7]. Biomaterials such as tricalcium phosphate and tricalcium silicates have also been suggested as additives to restorative materials to help prevent the demineralization cascade and subsequent collagen degradation [8,9].

The dentin matrix is penetrated by hollow dentinal tubules encapsulated by intratubular dentin. If the dentin, which is mineral-rich and collagen-poor, is demineralized by acids, both peritubular and intratubular mineral are removed, thereby enlarging the tubule diameters and revealing the fibrillar nature of the collagen matrix. The tubules are filled with dentinal fluid that is saturated with calcium and phosphate ions such as other extracellular fluids [10,11]. Thus, dentin is permeable, which is probably an important factor determining pulp reactions to caries and operative procedures [12]. According to Pashley et al. [13], dentin permeability affects the adhesive layer and may decrease bond strength. When the total-etch approach is used, acid etching with phosphoric acid etchant removes hydroxyapatite crystals from the dentin and increases the permeability of demineralized dentin. The use of total-etch dentin adhesives may result in higher permeability than self-etch dentin adhesives [14]. As a resolution, remineralization of demineralized dentin has been reported to reduce dentinal fluid flow [15,16].

Bioactive glass (BAG) was invented by Dr. Larry Hench and is used as a biomedical material for hard tissue regeneration. In contrast to inert glasses, BAG can induce remineralization by ion exchange and form a hydroxycarbonate apatite (HCA) layer [17]. It exhibits excellent biocompatibility [18], induces cell growth and odontogenic differentiation, and forms biomimetic remineralized tissue [19,20]. Recently, BAG has been utilized to remineralize enamel and dentin through direct application [21,22,23]. Further studies have focused on incorporating BAG into various dental materials. As a filler component of resin composites, BAG releases calcium and silica ions [24], displays antibacterial behavior [25,26], inhibits matrix metalloproteinase (MMP) activity [27], and can remineralize enamel [28] and dentin [29] without negatively affecting the mechanical properties of the parent material [30]. A resin cement containing BAG was reported to reduce the amount of MMP1 and MMP2 in dentin collagen, which affects dentin remineralization [9]. When incorporated into a glass ionomer, it was found to induce mineral formation on the surface of the material and underlying dentin [31].

Taken together, the results of previous studies have demonstrated that BAG can maintain its original bioactivity in dental materials and contribute towards the remineralization of tooth structure [24,25,26,27,28,29,30,31]. Based on these advantages, we hypothesized that BAG-containing dentin adhesive could form hydroxyapatite (HA) crystals on the demineralized dentin and contribute to reduce dentin permeability. Therefore, this study was designed to evaluate the effect of a novel BAG-containing dentin adhesive on the permeability of demineralized dentin via the micro-tensile bond strength (μTBS) test, field emission scanning electron microscopy (FE-SEM), dentinal fluid flow (DFF) rate measurement, and confocal Raman spectroscopy.

## 2. Materials and Methods

### 2.1. Specimen Preparation

Thirty-six caries-free extracted human third molars were obtained using a protocol approved by the Institutional Review Board of Kyung Hee University Dental Hospital (KHD IRB 1701-1). The coronal enamel was removed using a high-speed diamond saw (Isomet 5000; Buehler Ltd., Lake Bluff, IL, USA) to form a flat dentin surface under continuous water cooling. The surface was polished with 180-, 320-, and 600-grit silicon carbide (SiC) paper to produce a standard smear layer.

### 2.2. Material Preparation

#### 2.2.1. Synthesis of BAG

BAG 85S (85 mol% SiO_2_, 15 mol% CaO) was prepared without amination as described by Lee et al. [32]. All the reagents were purchased from Sigma-Aldrich (St. Louis, MO, USA). Briefly, it was prepared by sol-gel synthesis using a mixture of a precursor (calcium nitrate tetrahydrate) with deionized water (DW), co-solvents (ethanol and 2-ethoxyethanol), surfactant (hexadecyltrimethylammonium bromide; CTAB), and a catalyst (aqueous ammonia) at room temperature. After the mixture was stirred for 30 min, tetraethyl orthosilicate was added at a molar ratio of 85:15 (Si:Ca). Next, the solution was stirred for 4 h to form a gel-state solution. Then, a precipitate obtained from the gel-state solution was filtered, washed, and dried in air for 24 h. Next, the solution was heated to remove CTAB. After calcination at 600 °C in air for 5 h, the precipitate was washed with ethanol and DW, and the nanoparticles were dried under vacuum to obtain BAG.

#### 2.2.2. Dentin Adhesive Preparation

Table 1 shows the formulations of the prepared adhesives used in this study. All the reagents were also purchased from Sigma-Aldrich. The monomers were diurethane dimethacrylate (UDMA) and 2-hydroxyethyl methacrylate (HEMA). Camphorquinone and ethyl-4-(dimethylamino) benzoate (EDMAB) were added as the photoinitiator systems. As an antioxidant, 2,6-di-tert-butyl-4-methylphenol (BHT) was added. For the control dentin adhesive, 3 wt% of inert non-silanated silica was added (silica-containing adhesive, SCA). For the experimental group, BAG 85S was added at a concentration of 3 wt% to form the experimental dentin adhesive (BAG-containing adhesive, BCA).

### 2.3. Micro-Tensile Bond Strength (μTBS) Test

Ten teeth were assigned to undergo the μTBS test and were randomly divided into two groups according to the type of adhesive: SCA and BCA. The fresh, superficial dentin surface was exposed to a high-speed diamond saw (IsoMet 5000; Buehler, Lake Bluff, IL, USA). Then, each dentin surface was etched with 37% phosphoric acid gel (Etch-37; Bisco, Schaumburg, IL, USA) for 15 s, after which it was rinsed and blot dried. Each adhesive was applied to dentin for 15 s and gently air-dried. Then, the adhesive was light-cured for 20 s using an LED curing unit (Bluephase G2; Ivoclar Vivadent, Schaan, Liechtenstein) emitting 1200 mW/cm^2^. A resin composite (Any-Com; MEDICLUS, Cheongju, Korea) was incrementally built up to a thickness of 2 mm to a height of 4 mm. Each increment was light-cured for 20 s, with light placed directly over the composite. The specimens were stored in distilled water at 37 °C for 24 h.

After storage, each composite-tooth specimen was sectioned occluso-gingivally into 1.0 mm thick serial slabs using a high-speed diamond saw (IsoMet 5000). These slabs were further sectioned into composite-dentin beams (1.0 × 1.0 mm^2^). Forty composite dentin beams were produced in each group. These beams were divided into two sub-groups and stored in distilled water for 24 h (n = 20). No treatment was performed on the first 20 beams in the immediate group. The other 20 beams were accelerated-aged in 10 % sodium hypochlorite (NaOCl) for 1 h before testing [33]. The prepared beams were mounted on a testing jig with a cyanoacrylate adhesive (Zapit; Dental Ventures of America, Corona, CA, USA). The μTBS test was performed with a universal testing machine (AGS-X STD; Shimadzu, Kyoto, Japan) at a crosshead speed of 1.0 mm/min.

### 2.4. FE-SEM of the Adhesive Surface 

FE-SEM analysis was performed to evaluate the cured-surface changes of the SCA and BCA. A total of 10 resin composite blocks (Any-Com) were prepared with a silicone mold (6.0 × 6.0 × 4.0 mm^3^). The blocks were randomly divided into two groups (n = 5). A 37% phosphoric acid gel (Etch-37) was applied for 20 s on the surface of the block and rinsed with water. Then, either SCA or BCA was applied and light-cured for 20 s.

The specimens were immersed in simulated body fluid (SBF) for 2 weeks, and the solution was changed every 2 days to prevent autogenous precipitation. SBF was prepared according to the method of Tas et al. [34]. The composition of SBF solution with 27 mM HCO_3_^−^ is presented in Table 2. After storage, the surface of the adhesive on the resin composite block was thoroughly rinsed with distilled water for 3 min. The blocks were then treated as described by Perdigao et al. [35]. They were subsequently examined using FE-SEM (S-4700; Hitachi, Tokyo, Japan) at 10 kV.

### 2.5. FE-SEM of Dentin Surface

Ten teeth were assigned and randomly divided into two groups (SCA and BCA, n = 5). To simulate demineralized dentin, each dentin surface was etched with 37% phosphoric acid gel (Etch-37) for 60 s. Ten composite blocks (Any-Com) were fabricated, and each dentin adhesive was applied and light-cured, as previously described in Section 2.4. The adhesive-applied surface of the composite block was approximated to the demineralized dentin surface and then fixed with a flowable resin (G-Fix; GC, Tokyo, Japan). The composite-tooth specimens of the two groups were then stored in SBF solution at 37 °C for 2 weeks. The SBF was changed every 2 days to prevent autogenous precipitation. After storage, the composite block was removed from the specimen, and the dentin surface was thoroughly rinsed with distilled water for 3 min. The FE-SEM analysis was performed on the dentin surface, as described in Section 2.4.

### 2.6. Dentinal Fluid Flow (DFF) Rate Measurement

**Figure 1:**
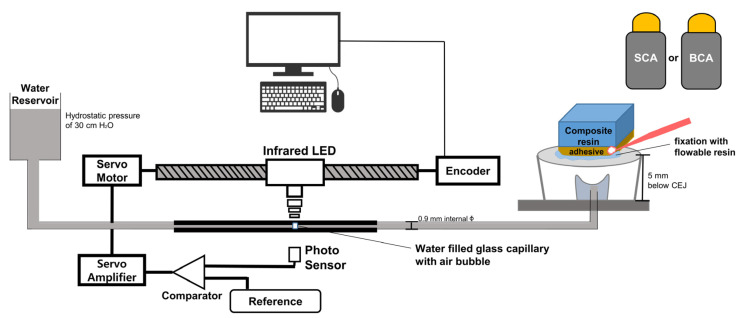
Schematic diagram of the real-time dentinal fluid flow (DFF) rate measurement device. The prepared specimen was connected to a glass capillary filled with water containing an air bubble. Abbreviations: SCA, silica-containing adhesive; BCA, BAG-containing adhesive; CEJ, cemento-enamel junction; LED, light-emitting diode.

Five teeth were used for real-time DFF rate analysis for each experimental group (n = 5). A schematic diagram of the DFF-rate analysis device is shown in Figure 1. The root portion of each tooth was removed 5 mm below the cemento-enamel junction using a high-speed diamond saw (Isomet 5000). The pulp tissue was completely removed using endodontic files and tissue forceps. Then, the tooth specimen was mounted on an acrylic plate with a hole drilled in its center. A metal tube that was 0.9 mm in diameter was inserted into the hole, ending within the pulp chamber. The acrylic with the tooth specimen was fixed, and the remaining exposed root surface was completely sealed with dentin adhesive (All-Bond Universal; Bisco) and flowable resin composite (Gaenial-Flow Universal; GC). This was then stored for 24 h in distilled water. Subsequently, each specimen was connected to a water reservoir under a hydrostatic pressure of 30 cm H_2_O to simulate the physiological pressure of the pulp tissue [36]. Each specimen underwent a stabilization procedure for 10 min after being connected to a subnanoliter-scaled fluid flow measuring device (NanoFlow; IB Systems, Seoul, Korea) [37].

A real-time DFF rate analysis was performed continuously for 5 min before acid etching to measure the baseline flow rate ($DFF_{Base}$). Acid etching was then conducted for 60 s. Then, the specimen was rinsed with distilled water and blot-dried. The same analysis was performed for 5 min to measure the pre-application flow rate through dentin simulating demineralization ($DFF_{Pre}$). After measuring the $DFF_{Pre}$, each tooth was approximated to the composite block where one of the adhesives (SCA or BCA) was cured on its surface and fixed as described in Section 2.5. They were then stored in the SBF solution at 37 °C for 2 weeks, with the solution being changed every 2 days to prevent autogenous precipitation. After storage, the composite block was removed from the specimen and thoroughly rinsed with distilled water for 3 min, and DFF rate analysis was then performed for 5 min to measure the post-application flow rate ($DFF_{Post}$). The real-time changes in the DFF rate were calculated by:

(1) $$\Delta DFF\;\left(\%\right)=\left(DFF_{Post}-DFF_{Pre}\right)\;÷\;DFF_{Pre}\times 100$$

### 2.7. Confocal Raman Spectroscopy

Six teeth were selected and divided into two groups (SCA and BCA). They were prepared as described in Section 2.5. To identify the chemical changes, we used a confocal Raman spectroscopy system (UniDRON, Yongin, Korea) with a 785-nm diode laser with 100 mW power and a 10× objective lens with 0.25 NA. With this system, Raman spectra were obtained three time points (before demineralization, after demineralization, and after SCA or BCA application) at 25 random locations (n = 75 per each time point) within a spectral range of 400–1100 cm^−1^, spectral resolution of 2 cm^−1^, and an acquisition time of 10 s.

### 2.8. Statistical Analysis

To identify differences in μTBS according to dentin adhesive and accelerated aging, two-way analysis of variance (ANOVA) was performed. To analyze DFF rate according to measurement time and ΔDFF of dentin adhesives, one-way ANOVA was conducted. The Bonferroni test was used for post-hoc analysis. The *p*-value was estimated using an R-squared approximation for degrees of freedom (α = 0.05).

## 3. Results

### 3.1. Micro-Tensile Bond Strength (μTBS) Test

The μTBS results are presented in Table 3. There was no difference in the μTBS of the SCA and BCA groups in either immediate or accelerated aging (*p* > 0.05). However, the μTBS of each adhesive significantly decreased after accelerated aging (*p <* 0.05).

### 3.2. FE-SEM of the Adhesive Surface 

**Figure 2:**
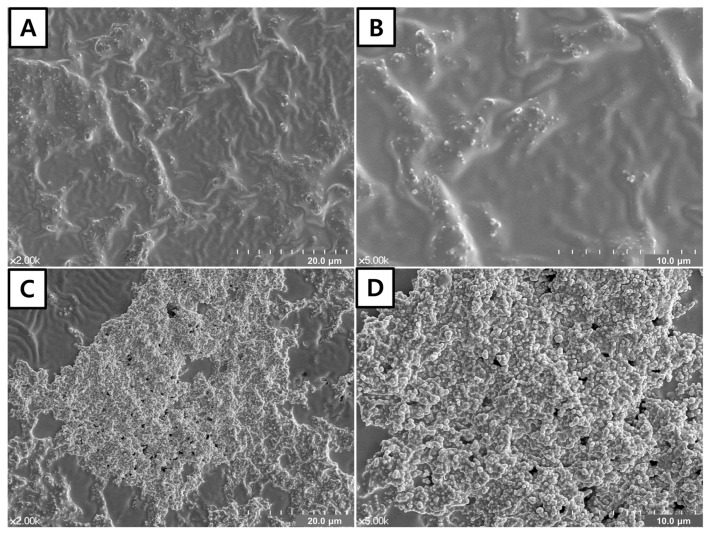
FE-SEM of the adhesive surface. (**A**,**B**). Adhesive surface of the SCA group with low and high magnification, respectively. No precipitates were observed on the surface of the SCA group. (**C**,**D**)**.** Adhesive surface of BCA group with low and high magnification, respectively. Multiple aggregations of the precipitates were observed.

Representative FE-SEM images of the SCA and BCA groups are shown in Figure 2. There were no precipitates on the surface of the SCA group (Figure 2A,B), whereas a bunch of precipitates were observed on the surface of the BCA group (Figure 2C). In the magnified image, small particles with a diameter of 200 nm were observed (Figure 2D).

### 3.3. FE-SEM of the Dentin Surface

**Figure 3:**
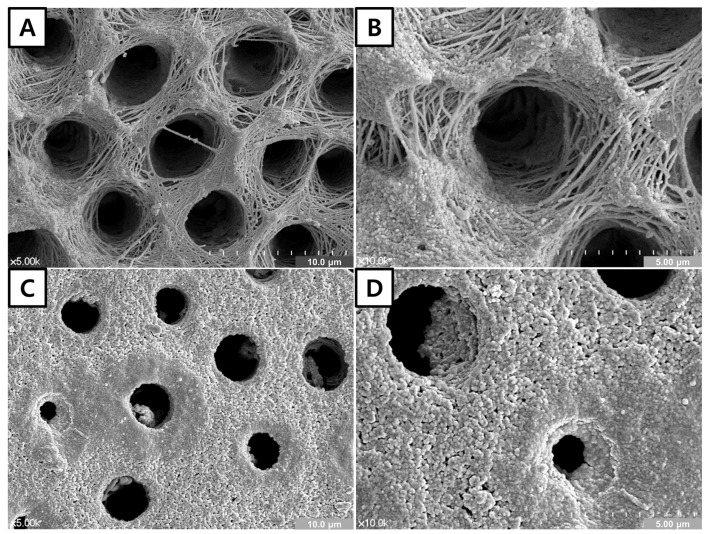
FE-SEM of the dentin surface. (**A**). Low magnification view (×5000). (**B**). High magnification view (×10,000) of SCA-approximated dentin surface. The exposed collagen network can be seen on the demineralized dentin surface. Some precipitates covering the dentin surface can be seen. (**C**). Low magnification view (×5000). (**D**). High magnification view (×10,000) of BCA-approximated dentin surface. The demineralized dentin surface was predominantly covered with small precipitates, and precipitates were observed inside the dentinal tubules, which obstructed dentinal tubule orifices.

Representative FE-SEM images are shown in Figure 3. In the SCA group, a demineralized dentin surface with open dentinal tubules was apparent (Figure 3A). The demineralized collagen network was clearly observed around the dentinal tubules, especially in the magnified image (Figure 3B). Some precipitates appeared around the dentinal tubules. However, the dentin surface was predominantly covered with small precipitates in the BCA group (Figure 3C), and many precipitates were observed in the dentinal tubules, which partially occluded dentinal tubules (Figure 3D).

### 3.4. Dentinal Fluid Flow (DFF) Rate Measurement

**Figure 4:**
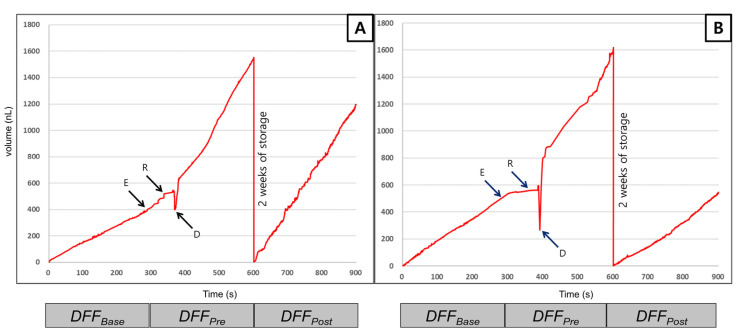
Representative graph of the real-time dentinal fluid flow (DFF) rate measurement. (**A**) The DFF rate of the SCA group. (**B**) The DFF rate of the BCA group. The gradient of each section means the DFF rate. The DFF rates of both groups decreased after being blot dried and increased more than that before acid etching. Abbreviations: E, etching with phosphoric acid; R, rinse; D, dry; DFF, dentinal fluid flow; SCA, silica-containing adhesive; BCA, BAG-containing adhesive.

The DFF values of both experimental groups are shown in Table 4, and the graph of real-time DFF rate analysis is shown in Figure 4. $DFF_{Base}$ values for the SCA and BCA groups were 1.38 and 1.79 nL/s, respectively. After demineralization, the $DFF_{Pre}$ values of the groups increased to 3.68 and 3.71 nL/s, respectively. Although both adhesives showed similar $DFF_{Base}$ and $DFF_{Pre}$ values, their $DFF_{Post}$ values were different. The DFF rate slightly increased by 6.54% in the SCA group but dramatically decreased by 50.10% in the BCA group. As shown in Figure 4, the DFF rates of both groups temporarily decreased at blot dry and increased gradually after etching. The DFF changes in both groups were similar before the indirect approximation of the two adhesives. While the $DFF_{Post}$ of the SCA group did not change after adhesive approximation, that of the BCA group decreased considerably.

### 3.5. Confocal Raman Spectroscopy

**Figure 5:**
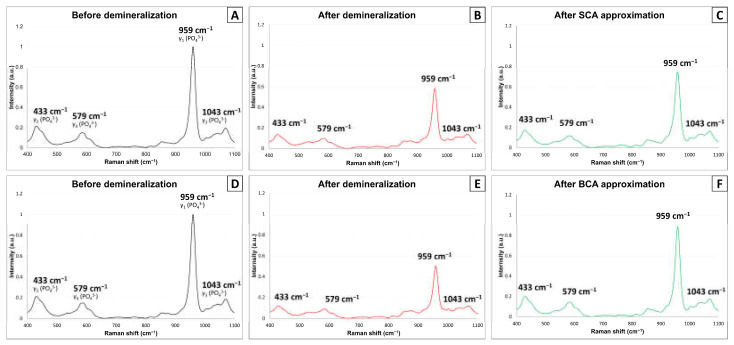
Confocal Raman spectra of the SCA and BCA group. (**A**–**C**) Raman spectra of the SCA group (before, after demineralization, and after SCA approximation, respectively). (**D**–**F**) Raman spectra of the BCA group (before, after demineralization, and after BCA approximation, respectively). Abbreviations: SCA, silica-containing adhesive; BCA, BAG-containing adhesive.

Figure 5 shows the representative changes in the Raman spectra of the SCA and BCA groups (n = 6). There were four different phosphate ion peaks (433, 579, 959, and 1043 cm^−1^) in both groups before demineralization (Figure 5A,C). The most intense peak was at 959 cm^−1^, which decreased dramatically after demineralization in both groups (Figure 5B,E). In addition, the intensities of another three peaks slightly decreased. After the approximation of both adhesives, these peaks slightly increased in the SCA group (Figure 5C). On the other hand, the intensity of peak at 959 cm^−1^ increased considerably in the BCA group (Figure 5F).

## 4. Discussion

Remineralization of dentin is an important issue because it can enhance tooth structure and further prevent collagen degradation. Although various methods have been suggested to induce tooth remineralization, most have only been possible in in vitro situations. For dentin remineralization agents to be clinically useful, the underlying demineralized dentin must be directly contacted. Thus, dentin adhesive is an ideal material for the incorporation of a remineralization agent. This study evaluated BAG-containing dentin adhesive using various methods and proved that it could partially remineralize adjacent dentin, thus reducing the permeability of demineralized dentin while maintaining bond strength.

BAG 85S was prepared using a sol-gel process. It has smaller particle size (160 nm) and larger surface area (423 m^2^/g) than conventional, melt-quenching derived BAG. Vollenweider et al. [23] compared the remineralization ability of sol-gel and melt-quenching derived BAG and showed that nano-sized BAG was far more efficacious than micrometer-sized BAG for remineralization. The surface area of BAG is important for HCA layer formation because ion release only occurs on its surface. Thus, the nanostructured BAG synthesized in the present study has sufficient potential for dentin remineralization. The formation of the HCA layer on the BAG surface plays an important role in the remineralization process. HCA is considered a precursor of hydroxyapatite (HA), which is thought to interact with the exposed collagen fibers to promote tissue mineralization [38]. When BAG is exposed to body fluids, there is an ion exchange from the fluids, forming silanol bonds on the surface of BAG and eventually a silica (SiO_2_)-rich layer. When exposed to increased pH, the Si–O–Si bonds of the silica-rich layer are disrupted, and the layer absorbs calcium ions (Ca^2+^) from the glass. This allows the glass surface to react with phosphate ions [(PO_4_)^3−^] to form an amorphous HCA layer. Over time, the amorphous HCA layer crystallizes into the HA layer.

Some dentin adhesives contain silanized inorganic fillers. The silanization of inorganic fillers plays an important role in chemical bonding to the organic matrix, thus reinforcing the mechanical properties of the adhesive [39]. However, it was thought that incorporation of BAG 85S might adversely affect dentin bond strength because it was not silanized. Although silanization of inorganic filler is necessary to chemically combine the organic matrix and inorganic filler, silanization of BAG may reduce its ion release ability by increasing its hydrophobicity. According to the results of the μTBS test, those of the SCA and BCA groups were not significantly different in both the immediate and accelerated-aged modes. These results indicate that the incorporation of BAG into the adhesive did not adversely affect the adhesion performance, which is consistent with the results of a previous study [27]. A common point of two studies was that the content of BAG was low (3% in this study, and 0.5–2% in Jun et al. [27], respectively).

For accelerated aging of the specimens, NaOCl storage was used in this study. Using this method, unprotected, exposed collagen fibrils after acid etching are deproteinized, which reduces bond strength [33]. It was selected to confirm whether the incorporated BAG hindered adhesive infiltration due to its particle size (160 nm). The interfibrillar spaces of demineralized dentin after etching have been reported to be within 20 nm [40] thus, there was a potential concern with adhesive infiltration. However, the μTBS values of both groups were not significantly different from each other after accelerated aging. This means that the BAG 85S used in this study did not hamper adhesive infiltration.

To evaluate the surface changes of SCA and BCA, the cured surfaces of both dentin adhesives were analyzed using FE-SEM. Although there were no precipitates on the surface of SCA, cluster-like precipitates were obvious on the dentin treated with BCA (Figure 2). It was assumed that the precipitates were part of the HCA layer formed on the surface of the BAG. This formation of precipitates was also reported in our previous study [31]. To identify the effect of precipitates on demineralized dentin, the dentin surface was also analyzed. The reason for observing the top surface of dentin without direct application of the adhesive was that it was technically impossible to physically remove only the thin adhesive and hybrid layers without damaging the underlying demineralized dentin. This experimental method was also utilized in other studies that evaluated the effect of a BAG-containing glass ionomer cement and composite resin [31,41]. There were more precipitates on the BCA-approximated dentin surface than on the SCA-approximated one. Theoretically, it was expected that there would not be any precipitates on the dentin surface of the SCA group. However, the FE-SEM analysis revealed some precipitates on the SCA-approximated dentin surface. It was attributed to the effect of storage solution. In this study, a 27 mM HCO_3_^−^ Tris SBF was used for specimen storage, as its chemical composition is more similar to human blood plasma than the original SBF prepared by Kokubo and Takadama [42], and is more proper in simulating dentinal tubule fluid [34]. However, it may also form autogenous calcium phosphate precipitates in the specimen due to supersaturation [43]. Thus, we changed the SBF every 2 days to prevent the formation of undesirable precipitates. Our previous study revealed that the difference between artificial saliva and 27 mM HCO_3_^−^ Tris SBF did not affect the dentin remineralization with every 2 days change [41]. However, this study showed different result in the FE-SEM analysis. It should be investigated further.

The indirect approximation of SCA and BCA to demineralized dentin was used in this study. It was due to the exclusion of the effect of adhesive resin, which could seal the exposed dentinal tubule. This indirect method has been used by Kim et al. [31] and Jang et al. [41] successfully. Considering the results of the FE-SEM analysis, the DFF rates of both adhesives were expected to decrease. However, the DFF rate of the SCA group increased by 6.54%, indicating that the precipitates did not contribute to the reduction of DFF rate. In the FE-SEM analysis, dentinal tubules were not occluded by precipitates (Figure 3A,B). However, the DFF rate was reduced by 50.10% in the BCA group, suggesting that the formed precipitates were HA crystals and might contribute to tubule occlusion (Figure 3C,D). Considering the remarkable reduction in DFF rate in the BCA group, it is assumed that formed precipitates may exist in deeper areas of the dentinal tubule.

Reducing the dentin permeability is important for dentin adhesion. Increased permeability may cause rapid degradation of the adhesive layer via continuous fluid movement, thus eventually deteriorating dentin adhesion [44]. As mentioned in the Introduction, BAG has the potential to remineralize dentin. This study not only proved its remineralization ability to dentin, but also reduced dentin permeability. The reduction in permeability was attributed to the formation of HA crystals inside the dentinal tubules. Considering these abilities of BAG, a synergistic reduction effect is expected if BCA is directly applied to demineralized dentin.

The chemical properties of the precipitates were analyzed by confocal Raman spectroscopy. Four distinct peaks (433, 579, 959, and 1043 cm^−1^), which are representatives for HA crystal were investigated. They refer to (PO_4_)^3−^(_ν__2_), (PO_4_)^3−^(_ν__4_), (PO_4_)^3−^(_ν__1_), and (PO_4_)^3−^(_ν__3_) respectively. The peak of 959 cm^−1^ was most sensitive to mineral change of the dentin. This result is consistent to that of Sauro et al. [45] and Marin et al. [46]. The SCA group showed a slightly increased HA crystal peaks after the approximation of the adhesive in spite of the absence of BAG incorporation. It may be attributed to autogenous calcium phosphate precipitates on the dentin surface observed in the FE-SEM images of this group. The precipitates in the BCA group showed more intense peaks than those in the SCA group. This suggests that the BCA approximated dentin surface was partly remineralized by newly formed HA crystals. This conclusion is also supported by the FE-SEM results. Because this study only investigated phosphate peaks to identify the formation of HA crystals, it was not possible to evaluate the quality of dentin remineralization. Two analysis of Raman parameters have been suggested. The measurement of mineral-to-matrix ratio between 960 and 1650 cm^−1^ can represent the volumetric fraction of mineral with respect to collagen [47]. Full width at half maximum (FWHM) at the peak of 959 cm^−1^ can also reveal the degree of crystallinity and defects in the HA crystals [48,49].

In addition, further studies for the changes of mechanical properties such as microhardness, nanoindentation, or elastic modulus measurement, should be performed to clarify the remineralization effect of BAG. These methods can provide the proof of intra-fibrillar remineralization of the dentin. Micro-hardness measurement about elastic modulus measurement of BAG-containing dentin adhesive and BAG-containing resin composite also reported the possibility of dentin remineralization [29,41].

## 5. Conclusions

Within the limitations of this study, BAG-containing dentin adhesive reduced the permeability of demineralized dentin via its remineralization potential while maintaining bond strength to dentin. This study provides reliable evidence for incorporating BAG into dentin adhesive as a multifunctional agent.

## Figures and Tables

**Table 1:** The chemical compositions of the control and experimental adhesives.

Material	Content (wt.%)	
	SCA	BCA
UDMA (Diurethane dimethacrylate)	42.8	42.8
HEMA (Ethylene glycol methacrylate)	12.2	12.2
CQ (Camphorquinone)	0.5	0.5
EDMAB (ethyl-4-dimethylamino benzoate)	1.0	1.0
BHT (2,6-di-tert-butyl-4-methylphenol)	0.25	0.25
TP (2,2′-(P-tolylimino)-diethanol)	0.25	0.25
Ethanol	40.0	40.0
BAG 85S	-	3.0
Silica	3.0	-

Abbreviations: BAG, bioactive glass; SCA, silica-containing adhesive; BCA, BAG-containing adhesive.

**Table 2:** The composition of 27 mM HCO_3_^−^ Tris simulated body fluid (SBF).

Storage Solution	Composition (Amount, g/L)
27 mM HCO_3_^−^ Tris SBF	NaCl (6.547) NaHCO_3_ (2.268) KCl (0.373) Na_2_HPO_4_∙2H_2_O (0.178) MgCl_2_∙6H_2_O (0.305) CaCl_2_∙2H_2_O (0.368) Na_2_SO_4_ (0.071) (CH_2_OH)_3_CNH_2_ (6.057)

**Table 3:** Micro-tensile bond strengths of both experimental groups (n = 20, unit: MPa).

Adhesive	Immediate	Aged
SCA	29.66 ± 7.71 ^a^	18.91 ± 8.63 ^b^
BCA	32.47 ± 11.56 ^a^	21.68 ± 6.93 ^b^

The different lower-case letters indicate statistical significance in both row and columns (*p* *<* 0.05). Abbreviations: SCA, silica-containing adhesive; BCA, BAG-containing adhesive.

**Table 4:** Average dentinal fluid flow (DFF) rate and reduction rate (n = 5).

Adhesive	DFF Rate (nL/s)			ΔDFF
	$DFF_{Base}$	$DFF_{Pre}$	$DFF_{Post}$	(%)
SCA	1.38 ± 0.65 ^A^	3.68 ± 0.44 ^B^	3.91 ± 0.58 ^C^	+ 6.54 ^a^
BCA	1.79 ± 0.26 ^A^	3.61 ± 0.14 ^B^	1.80 ± 0.12 ^C^	−50.10 ^b^

Abbreviations: SCA, silica-containing adhesive; BCA, BAG-containing adhesive; DFF, dentinal fluid flow. Different capital letters indicate the significant statistical difference in each row. Different lower case letters indicate the significant difference in each column.

## Data Availability

The data presented in this study are available on request from the corresponding author.
